# Viral metagenomics of aphids present in bean and maize plots on mixed-use farms in Kenya reveals the presence of three dicistroviruses including a novel Big Sioux River virus*-*like dicistrovirus

**DOI:** 10.1186/s12985-017-0854-x

**Published:** 2017-10-02

**Authors:** Francis O. Wamonje, George N. Michuki, Luke A. Braidwood, Joyce N. Njuguna, J. Musembi Mutuku, Appolinaire Djikeng, Jagger J. W. Harvey, John P. Carr

**Affiliations:** 1 Department of Plant Sciences, University of Cambridge, Cambridge, CB2 3EA UK; 2 International Livestock Research Institute, 30709 Naivasha Road, Nairobi, Kenya; 3 Biosciences eastern and central Africa-International Livestock Research Institute (BecA-ILRI) Hub, Nairobi, 30709-00100 Kenya; 4 Present Address: The Africa Genomics Center and Consultancy, Nairobi, Kenya; 5 Present Address: Centre for Tropical Livestock Genetics and Health, The Roslin Institute & Royal (Dick) School of Veterinary Studies, Easter Bush, Edinburgh, Midlothian EH25 9RG UK; 6 Present Address: The Feed the Future Innovation Lab for the Reduction of Post-Harvest Loss, Kansas State University, Manhattan, KS 66506 USA

**Keywords:** Metagenomics, Phylogenetics, Recombination, Dicistrovirus, Potyvirus, Vector, Aphid, Epidemiology

## Abstract

**Background:**

Aphids are major vectors of plant viruses. Common bean (*Phaseolus vulgaris* L.) and maize (*Zea mays* L.) are important crops that are vulnerable to aphid herbivory and aphid-transmitted viruses. In East and Central Africa, common bean is frequently intercropped by smallholder farmers to provide fixed nitrogen for cultivation of starch crops such as maize. We used a PCR-based technique to identify aphids prevalent in smallholder bean farms and next generation sequencing shotgun metagenomics to examine the diversity of viruses present in aphids and in maize leaf samples. Samples were collected from farms in Kenya in a range of agro-ecological zones.

**Results:**

*Cytochrome oxidase 1* (*CO1*) gene sequencing showed that *Aphis fabae* was the sole aphid species present in bean plots in the farms visited. Sequencing of total RNA from aphids using the Illumina platform detected three dicistroviruses. Maize leaf RNA was also analysed. Identification of *Aphid lethal paralysis virus* (ALPV), *Rhopalosiphum padi virus* (RhPV), and a novel Big Sioux River virus (BSRV)-like dicistrovirus in aphid and maize samples was confirmed using reverse transcription-polymerase chain reactions and sequencing of amplified DNA products. Phylogenetic, nucleotide and protein sequence analyses of eight ALPV genomes revealed evidence of intra-species recombination, with the data suggesting there may be two ALPV lineages. Analysis of BSRV-like virus genomic RNA sequences revealed features that are consistent with other dicistroviruses and that it is phylogenetically closely related to dicistroviruses of the genus *Cripavirus*.

**Conclusions:**

The discovery of ALPV and RhPV in aphids and maize further demonstrates the broad occurrence of these dicistroviruses. Dicistroviruses are remarkable in that they use plants as reservoirs that facilitate infection of their insect replicative hosts, such as aphids. This is the first report of these viruses being isolated from either organism. The BSRV-like sequences represent a potentially novel dicistrovirus infecting *A. fabae*.

**Electronic supplementary material:**

The online version of this article (10.1186/s12985-017-0854-x) contains supplementary material, which is available to authorized users.

## Background

Aphids are important insect pests of plants and vectors for plant-infecting viruses [1, 2]. Aphids are themselves infected by viruses, which may cause disease in the insects [1, 3]. For this reason, aphid-infecting viruses have been investigated with respect to their potential use in biological control or as agents that might interfere with aphid-mediated transmission of phytopathogenic viruses [4, 5]. However, viruses can sometimes act as mutualists, as demonstrated in at least one case for aphid-infecting viruses by a DNA virus, the *Densovirus Dysaphis plantaginea virus*, which promotes production of winged forms of its host, the rosy apple aphid, which enhances dissemination of both the host and virus [6].

The *Dicistroviridae*, which are *Picorna*-like positive-sense RNA viruses, are among the best-studied pathogenic viruses of aphids [3]. *Aphid lethal paralysis virus* (ALPV) and *Rhopalosiphum padi virus* (RhPV) are members of the family *Dicistroviridae* and the genus *Cripaviridae* (type species, *Cricket paralysis virus*: CrPV) [7]. Studies of CrPV revealed two internal ribosome entry sites preceding each of the two non-overlapping open reading frames (ORFs) of the dicistronic RNA genome [8]. ORF1 encodes non-structural proteins: a suppressor of RNA silencing; a helicase; a protease; the viral genome linked protein (VPg), and the RNA-dependent RNA polymerase (RdRp). ORF2 encodes four structural proteins, VP1–4 [3]. ORFs 1 and 2 are translated from the genomic RNA, producing polyproteins that are cleaved to yield the mature viral proteins [9]. RhPV, first isolated from *R. padi* in 1981, decreases aphid longevity and fecundity [10, 11]. ALPV was isolated from *R. padi* in South Africa and shares many biophysical properties with RhPV [12, 13]. RhPV has a genome that is slightly larger (10Kb) than that of ALPV (9.8Kb) [14, 15]. Both RhPV and ALPV can be transmitted between insects (horizontal transmission) and transovarially (vertical transmission) [3]. Remarkably, these aphid-infecting viruses are also transmitted horizontally through plants, which serve as infection reservoirs but are not considered to be hosts, since they do not support virus replication [3]. *Big Sioux River virus* (BSRV) was first isolated from honeybees (*Apis mellifera*) following a study of the bee microbiome to identify potential causes of bee colony collapse disorder in the United States [16]. BSRV has also been isolated from mosquitoes (*Culex tritaeniorhynchus*) and in the soybean aphid (*Aphis glycines*) in China [17, 18]. Neither the diversity of aphids nor the range of viruses infecting them has been examined in East Africa. We carried out viral metagenomic studies on aphid and plant samples collected on smallholder farms in from different agroecological zones in Kenya, focusing on farms where common bean and/or maize were grown.

## Methods

### Aphid and maize sampling sites

**Fig. 1:**
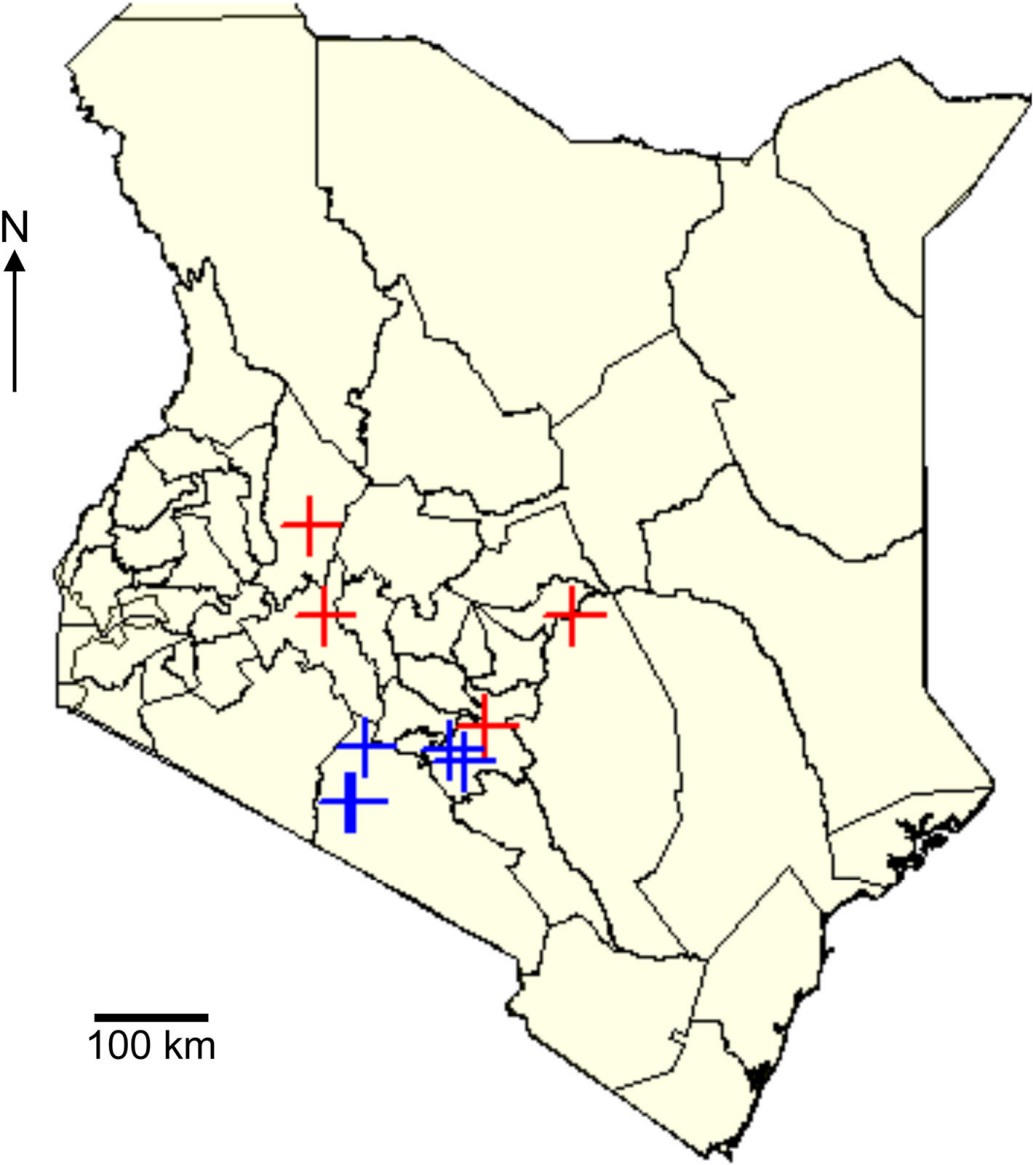
Sampling sites in Kenya**.** Crosses indicate sampling sites in the Central, Eastern and Rift Valley regions of Kenya where farmers’ fields were sampled for aphids (blue) and maize leaf (red). Black lines demarcate county boundaries. Three farms per site were sampled for aphids in Ndeiya, Kiambu county (humid, highland), Oloirien, Kajiado county (semi-arid, highland), Katumani, Machakos county (semi-arid, lowland) and Kaiti, Makieni county (Humid, lowland). For the maize samples, 14 farm sites in four counties were sampled for maize leaf samples. These were in Baringo (semi-arid, highland), Machakos (semi-arid, lowland), Kitui (semi-arid, lowland) and Nakuru (humid, highland). The details of the sampling sites and GPS coordinates are provided in Additional File 1: Table S1

The aphid samples were collected from a total of nine sites over four farms in four bean growing agro-ecological zones in Kenya based on different altitudes in meters above sea level (m asl) and known climatic conditions. These were Ndeiya (Humid, highland), Oloirien (semi-arid, highland), Katumani (semi-arid, lowland) and Kaiti (Humid, lowland) (Fig. 1). Apart from the Katumani farms where beans were grown exclusively, all other farms had beans intercropped with a variety of other food crops. Aphid samples were collected in November 2014, which coincided with the October–November planting season.

Maize leaf samples were collected from 14 sites in three counties in Kenya (Fig. 1). These were collected over the months of June–August from Baringo (semi-arid, highland), Machakos (semi-arid, lowland), Kitui (semi-arid, lowland) and Nakuru (humid, highland) (Additional file 1: Supplementary Table S1)**.**

### Trapping of aphids

To trap winged (alate) aphids, five yellow bowls (22 cm diameter) containing approximately 300 ml of 1% (*w*/*v*) SDS mixture were set up at the corners and the center of a 10x10m area within a bean plot. These traps were placed on stilts raised to the same height as the bean plants and left for two hours during the warmest part of the day (approximately 12:00 to 14:00 East Africa Time). Aphids were recovered from traps using plastic microbiological inoculation loops (VWR International bvba, Leuven, Belgium) and transferred individually to 1.5 ml micro-centrifuge tubes containing 500 μl of RNALater (Thermo Fisher Scientific, Waltham, Massachusetts, USA). For collection of wingless (apterous) aphids, bean plants within the proximity of the traps were selected at random and examined for wingless aphids for capture and preservation in RNALater. Aphid samples were transported in racks and stored at 4 °C for up to 4 days before processing. Apterous aphids were collected at all four sites (Fig. 1). Alate aphids were available only at the Ndeiya site (Fig. 1).

### DNA and RNA extraction from aphids

For nucleic acid extraction, ten wingless aphids were pooled together per sampling site into nine sample pools (P1-P9). The winged aphids were not pooled and were treated as individual samples (labelled as samples S16-S45) (Additional file 1: Table S1). Aphids were transferred to 2.0 ml screw-capped tubes, prefilled with 1.4-mm (diameter) ceramic beads for homogenizing using the MagNA Lyser® instrument (Roche, Basel, Switzerland). Lysis buffer (200 μl) was added to the tubes and the samples homogenized by shaking for 30 s at 7000 Hz. Of the supernatant, 120 μl was set aside for RNA extraction and 80 μl retained for DNA extraction. Semi-automated RNA and DNA extraction for RNAseq Illumina sequencing and *CO1* PCR identification was done using the MagNA Pure® LC RNA Isolation kit III and MagNA Pure® LC DNA Isolation Kit II (Tissue) respectively on the MagNa Pure LC instrument (Roche) following the manufacturer’s instructions. Extracted RNA and DNA was analysed for concentration and purity using a Nanodrop® ND1000 spectrophotometer (Thermo Scientific).

### *Cytochrome oxidase subunit 1* gene sequencing of individual aphid samples

*Cytochrome oxidase subunit 1* (*CO1*) gene sequencing using template DNA from individual aphids was conducted using the aphid-specific primers, Favret F and Favret R (sense 5′-ACC AGT TTT AGC AGG TGC TAT TAC-3′ antisense, 5′-GTA TAT CGA CGA GGT ATA CCA TTT-3′) using DNA from individual aphids as template (S16-S45). The primers bordered a 700-base pair (bp) region of the *CO1* gene [19]. Purified PCR products were sequenced in both directions by automated Sanger sequencing [20, 21].

### Library preparation for next generation sequencing of aphid samples

Thirty-nine libraries were prepared for sequencing using the Illumina MiSeq system. Of these libraries, nine consisted of pools of ten aphids taken from the area sampled. The remaining 30 were made from individual aphids caught in traps. Libraries were prepared using the TruSeq RNA v2 kit (Illumina, San Diego, CA) protocol as per the manufacturer’s instructions. Libraries were normalised, pooled and sequenced in two runs using the Illumina MiSeq system. Paired end reads were generated using the 2 × 250 cycle V2 kit on the MiSeq.

### RNA extraction from maize samples and next generation sequencing

RNA was extracted using Trizol (Ambion) as per manufacturer’s instructions. Ribosomal RNA (rRNA) depletion was performed with Ribo-Zero Magnetic Kits (Plant Leaf – Epicentre), and depletion confirmed on a pre-cast 6% TBE-urea gel (Novex, Life Technologies). Indexed stranded libraries were constructed using Scriptseq V2 RNA-Seq Library Preparation kits (Epicentre) and Scriptseq Index PCR primers (Epicentre). Purification steps were performed using Agencourt AMPure XP beads. Library quantity and quality were checked using Qubit (Life Technologies) and on a Bioanalyzer High Sensitivity DNA Chip (Agilent Technologies). Libraries were sent to Beijing Genomics Institute for 100 bp paired-end sequencing on one lane of a HiSeq 2000 (Illumina).

### Bioinformatics

The quality of reads generated was checked using FastQC (available online at: www.bioinformatics.babraham.ac.uk/projects/fastqc/). The reads were trimmed to attain optimum quality using the dynamic trim function of SolexaQA [22] and FASTX-Toolkit (http://hannonlab.cshl.edu/fastx_toolkit/). A de novo assembly of the reads was done using Trinity (http://trinityrnaseq.github.io/) based on the de Bruijn graph algorithm. The resulting contigs from de novo assembly were analysed by BLASTn against a virus database to detect all viral sequences present in the data. In the Virfind online analysis pipeline, the sequences were trimmed (default setting ~5 bases). De novo assembly was performed using Trinity (v.2.0.2) but where the average sequence length was ≤80 nucleotides, de novo sequence assembly was done by Velvet with kmer = 15, 19, 23, 27, to generate sequences ≥90 nucleotides. Where the average sequence length ≤ 40 nucleotides, de novo sequence assembly was done by Velvet with kmer = 11, 13, to generate sequences ≥90 nucleotides. Assembled contigs were subjected to a BLASTn search in NCBI at the default e-value (0.01) to generate annotated sequences. All sequences not detected as ‘virus’ by BLASTn were subjected to a BLASTx search against all GenBank virus proteins with the e-value at the default (0.01). For the maize samples, libraries were de-multiplexed allowing one error within the index sequence and de novo assembly done with Trinity using a custom script.

### Confirmation of presence of ALPV, RhPV and BSRV-like viruses from aphid and maize samples by reverse transcription-PCR (RT-PCR)

To confirm the presence of ALPV, RhPV and BSRV-like viruses in the pooled samples, cDNA was made by reverse transcribing 500 ng of total RNA extracted from aphids in Pools 1–9 using GoScript First Strand synthesis system (Invitrogen). Random hexamers were used as primers in a total volume of 20 μl and reverse transcription done at 42 °C for 50 min as recommended by the manufacturer. Primers for ALPV based on the KE Aphid P7 and KE Aphid P9 sequences were ALFWD1 5′-CAA AAC AAT ACA AAA TGTC-3′ and ALRV1 5′-GGT TTG TTT AAA ATC GTT GCC-3′ targeting a 511 bp region corresponding to the sequence positions 511 and 1022 in ORF1 of the ALPV genome. The primers for BSRV-like sequences were based on sequence S37 and were: BSRV1 FWD-5′-ACA ATT TAT ATC GTT TAG GTT-3′, and BSRV1 REV-5′-TTA CTA AGG TTT AAA TCT TTA-3′ amplifying a 511 bp region between (approximate 511–1015) in ORF1 of the S42 BSRV genome. For ORF2, primers BSRV2 FWD-5′-GTC AAA ACT AAA TTT CAT TCA-3′ and BSRV2 REV-5′-GG TGT AAT CAT GTG AAA TCT T-3′ were used targeting a 555 bp region (approximate positions 8467–9022). Primers for RhPV detection in aphid samples were RhPVF1 5′-GCA AAC TCA GTA TCT TCA GC-3′ and RhPVR1 5′-TTT GAT TTA TGG CGT GGT GG-3′, which have previously been used in RhPV detection [23]. For RhPV detection from maize samples, custom primers were designed from the assembled RhPV sequence from sample T2F2S4 (sequence available in Additional file 2: Text file S1). The two primer pairs RPV_200_F1–5′-TTT GGA AGA CGT GTG CGAGA-3′/ RPV_1100_R1–5′-TCG TGC AGC TG GAA CGAAT-3′ (approximate positions 228–1016) and RPV_1350_F1–5′-GAT GGG TAC ACT GGA CAG CC-3′/ RPV_ 2300_R1–5′-CTC TCG CTC GCA GCA AATTC-3′ (approximate position 1401–2236) targeted 789 bp and 836 bp long regions of the assembled sequence, respectively. The primer sequences and sequences from the amplicons generated are shown in the additional information (Additional file 3: Table S2).

For PCR, 1 μl of cDNA solution was used in the reaction using the various primers and Biomix Red® PCR premix (Bioline, London, UK) in a 20 μl reaction. Cycling conditions for all primers were 3 min at 94 °C followed by 35 cycles of 30s at 94 °C/ 30s at 55 °C/ 1 min at 72 °C and a final extension of 7 min 72 °C. PCR products were analysed on a 2% agarose gel in 1X TBE containing Gel Red and electrophoresed in 1X TBE buffer. PCR products were purified and sequenced in both directions by automated Sanger sequencing [20, 21] at the Biosciences eastern and central Africa Hub at the International Livestock Research Institute in Nairobi, Kenya or Source Biosciences (Cambridge UK).

### Phylogenetic and sequence analysis of ALPV and BSRV-like genomes

Multiple sequence alignments were done using MUSCLE [24] in Molecular Evolutionary Genetics Analysis version 6.0. (MEGA 6) [25]. The evolutionary history was inferred using either the Neighbor-Joining method [26]. ORF Finder (https://www.ncbi.nlm.nih.gov/orffinder/) was used for ORF prediction. To annotate the proteins encoded by the ORFs, an online search was conducted in the NCBI conserved domain database (https://www.ncbi.nlm.nih.gov/Structure/cdd/cdd.shtml) for the location of conserved domains.

### Pairwise comparisons for percentage similarity at nucleotide and protein levels

The percentage differences between the nucleotide sequences of the isolates was done by doing a multiple sequence alignment using MUSCLE in the Sequence Demarcation Tool software version 1.2 [27].

### Recombination analyses

Analyses to detect recombination was done using RDP4 software package. Alignments and analyses were performed for ORF 1 and ORF 2 separately. Default parameters were used for the RDP [28], GENCONV [29], MAXCHI [30], 3Seq [31], SiScan [32] and CHIMAERA [33] methods selected for the analysis. A Bonferroni corrected *p* value of 0.05 was considered significant. Evidence of recombination was accepted if it was supported in at least three different methods with a *p* value >10^−6^.

## Results

### Aphid species identification by *Cytochrome oxidase subunit 1* gene sequencing revealed that *Aphis fabae* was the only aphid species present in bean plots at sampled sites

Aphids were trapped in bean fields at the locations shown in Fig. 1. Nucleic acids were extracted from aphids for PCR-based aphid species identification and for Illumina sequencing of RNA. Following PCR of aphid DNA using primers specific for *CO1*, a basic local alignment search tool (BLAST) [34] search of the generated *CO1* gene sequences showed that the samples were all *A. fabae.* The *CO1* sequences generated from Sanger sequencing are available in the additional files (Additional file 4: Text S3). Confirmatory results were provided by analysis conducted using Virfind online bioinformatics platform (http://virfind.org/j/virfind-pipeline) [35]. This analysis yielded longer *CO1* gene sequence reads than from the automated Sanger sequencing and likewise identified the sequences as originating from *A. fabae* (Additional file 5: Text S4). A summary of results of the *CO1* gene identification using the BLAST tool using sequences from Sanger sequencing and from Virfind are shown in the additional files (Additional file 6: Table S3).

### Detection of dicistroviruses RNA sequences in aphid and maize samples

RNA isolated from aphids sampled in bean plots and isolated from maize leaves was subjected to Illumina sequencing. Two sequence analysis pipelines were used to analyse the sequence data from the pooled and individual aphid samples (see Materials and Methods). Results from analysis of the assembled sequences revealed the presence of three known or putative dicistroviruses: ALPV, RhPV, and a BSRV-like virus in both pooled and individual aphid samples. The samples where these viruses were detected are shown in Table 1.

**Table 1:** Aphid and maize samples containing dicistrovirus sequences

Sample	location	Source	Sequence length	GenBank ID	Genbank ID of closest match and annotation in GenBank		% match
P4	Oloirien	Aphid	10,226	KY933253	JF423195.1	Big Sioux River virus	87
P5	Oloirien	Aphid	10,213	KY933252	JF423195.1	Big Sioux River virus	87
P6	Oloirien	Aphid	9640	KY933251	JF423195.1	Big Sioux River virus	87
P7	Ndeiya	Aphid	9417	KY933250	JF423195.1	Big Sioux River virus	87
P9	Ndeiya	Aphid	9691	KY826434	JF423195.1	Big Sioux River virus	87
S42	Ndeiya	Aphid	9620	KY933254	JF423195.1	Big Sioux River virus	87
S37	Ndeiya	Aphid	10,281	KY933255	JF423195.1	Big Sioux River virus	87
S36	Ndeiya	Aphid	9690	KY933256	JF423195.1	Big Sioux River virus	87
S32	Ndeiya	Aphid	9655	KY933257	JF423195.1	Big Sioux River virus	87
S28	Ndeiya	Aphid	10,237	KY933258	JF423195.1	Big Sioux River virus	87
S19	Ndeiya	Aphid	9647	KY933259	JF423195.1	Big Sioux River virus	87
P7	Ndeiya	Aphid	9792	LN907588	JX480861.1	Aphid lethal paralysis virus	93
P9	Ndeiya	Aphid	9801	LN907586	JX480861.1	Aphid lethal paralysis virus	93
S45	Ndeiya	Aphid	9613	MF458893	JX480861.1	Aphid lethal paralysis virus	93
T1F4S3	Baringo	Maize	5459	n/a	KX883690.1	Aphid lethal paralysis virus	98
T1F5S2	Baringo	Maize	7231	n/a	KX883690.1	Aphid lethal paralysis virus	98
T2F3S4	Kitui	Maize	9828	MF458892	KX883690.1	Aphid lethal paralysis virus	97
T2F3S4	Kitui	Maize	6816	n/a	AF022937.1	*Rhopalosiphum padi* virus	82

**Fig. 2:**
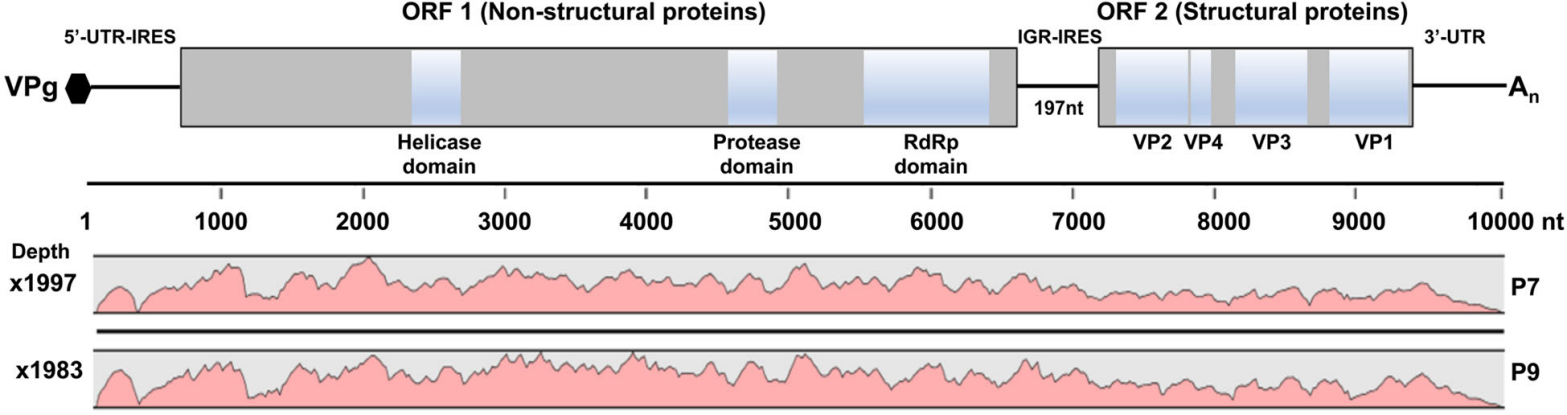
Genome organization and mapping coverage of *Aphid lethal paralysis virus* (ALPV) RNA sequences from samples P7 and P9. The positive-sense single-stranded RNA genome of ALPV has a length of approximately 9800 nucleotides (nt). A protein molecule (VPg: virus-protein-genome linked) is covalently attached to the 5′-end of the genomic RNA, which has a 3′-poly(A) tail indicated by A_n_. The 5′-untranslated region (5′-UTR) and the intergenic region (IGR) are 338 and 197 nt, respectively, and each function as an internal ribosome entry site (IRES) for translation of the two open reading frames (ORFs: gray boxes). ORF1 is 6111 nt long and is predicted to encode a 2037 amino acid precursor for the viral non-structural proteins. The ORF1 product contains the conserved motif for the 3C–like protease domain between residues 1395–1443 of the ORF1 product. Only putative functional domains in the virus-encoded proteins that can be predicted with a high degree of certainty are shown in the genome map (upper panel). Predicted helicase and RNA dependent RNA polymerase (RdRp) domains (indicated by blue boxes) were determined by searching for sequence homology in the NCBI conserved domain database. ORF2 is 2403 nt long and is translated into an 801 amino acid polyprotein that self-processes into four mature structural proteins VP2, VP4, VP3 and VP1 (indicated by blue boxes). The cleavage sites for the ALPV structural proteins have not yet been experimentally determined. However, their deduced positions based on amino acids alignments have been proposed [36]. By searching for the conserved motifs the cleavage position was determined for VP2/VP4 (I_228_ AATAQ/VGTEAI_238_) to be between residues 228–238 and VP3/VP1(I_553_ to RGVAQ/VNVAES_563_) to be between residues 553–563 of the amino acid sequence of ORF2 of ALPV. Sequence reads from samples P7 and P9 were mapped against an ALPV sequence from GenBank (reference NC004365) in CLC workbench version 5.1. The pink traces represent the depths of coverage at each nucleotide position which was × 1997 and × 1983 for samples P7 and P9, respectively

Three aphid samples (P7, P9 and S45) and sample T2F3S4 from maize had ALPV sequences approximately 9.6 Kb in length (Table 1). This genome size was comparable to other full-length ALPV genomes in GenBank (Table 2). Sequences from samples P7 and P9 were mapped against an ALPV genome from GenBank (reference number NC004365) in CLC workbench version 5.1. to get deeper sequence coverage (Fig. 2). The upper panel in Fig. 2 shows diagrammatically the arrangement of putative functional domains in the proteins encoded by the genome but showing only those predicted with highest confidence. These two sequences from aphid samples (P7 and P9) were renamed KE (Kenya) Aphid P7 and KE Aphid P9 while sample T2F3S4 from maize was renamed KE Maize in further analyses described in this article. ALPV sequences were also detected in two other maize samples (T1F4S3 and T1F5S2) but these were partial genomes and were not used in subsequent analyses. The ALPV sequences from these samples are available in the additional materials (Additional file 7: Text file S4).

**Table 2:** Geographic origin, isolation host and genome segment characteristics of the 8 ALPV isolates examined in this study

Isolate Name	GenBank No	Country of origin	Host/Reservoir	Genome size (bp)	reference
KE Maize	MF458892	Kenya	Maize	9749	This study
KE Aphid P7	LN907588	Kenya	*A. fabae*	9792	This study
KE Aphid P9	LN907586	Kenya	*A. fabae*	9801	This study
Israel	JX480861	Israel	*Aphis nerii*	9835	[53]
S. Africa	NC004365	South Africa	*R. padi*	9812	[15]
China	JQ320375	China	Bat	9819	[54]
Spain	JX045858	Spain	*Apis mellifera*	9327	[55]
USA	KJ817182	USA	*A. pisum*	9940	[39]
E. Timor	KX830963	East Timor	Aphid?	9789	[56]

One maize sample, T2F3S4, contained a 6816 bp sequence annotated as RhPV (Additional file 2: Text file S1). Additionally, there were shorter (~550 bp) assembled sequences annotated as RhPV from aphid samples (Additional file 8: Text file S5). By comparison, the full-length RhPV genomes available in GenBank are 10Kb in length. For the purposes of this study, RhPV sequences were not analysed further except by confirming the presence of the viral RNAs using RT-PCR and sequencing of the DNA products.

Eleven samples had sequences ranging from 9640 to 10,281 nucleotides long annotated as ‘Big Sioux River virus’ following de novo assembly (Table 1). The variation in the length of the genomic RNA sequences was mainly due to partial sequence assembly of the 3′-untranslated region. Of the 11 sequences, sample S37 (GenBank number KY933255) was the longest assembly at 10,281 nucleotides long including a 15 nucleotide polyA tail and is most likely the most complete of the BSRV-like genomes assembled. Though GenBank contained no full-length genomes for BSRV to allow complete sequence comparisons, the available sequences were sufficient for authentication of the BSRV-like virus RNAs. A list of other virus-like sequences detected from the aphid samples following BLASTx are in the additional information (Additional file 9: Table S4).

### RT-PCR confirmed the presence of ALPV, RhPV and BSRV-like virus sequences in aphid and maize leaf samples from Kenya

We used RT-PCR and DNA sequencing to confirm the presence of the three putative dicistroviruses. For the aphid samples, cDNA templates were synthesized using RNA from samples P4, P5, P6, P7 and P9 and virus-specific primers used to confirm presence of the three viruses (Additional file 3: Table S2). PCR products generated using ALPV- and BSRV-specific primers from the pooled aphid samples migrated to approximately 500–550 bp on agarose gels, which was within the expected size range of the primers used (Additional file 10: Fig. S1). A BLAST search for homology confirmed that the amplified sequences had similarity to ALPV and BSRV-like viruses. The identity of RhPV was confirmed in maize samples by RT-PCR and Sanger sequencing (Additional file 3: Table S2; Additional file 11: Fig. S2). However, we were unable to confirm presence of RhPV from the aphid samples by RT-PCR using either the custom primers used to detect the virus in the maize sample or by primers used by other groups [23].

### Analysis of ALPV sequences

To predict the domains encoded by the ORFs, analysis of the ORF sequences was conducted in the NCBI conserved domain database. Results revealed the predicted regions coding for the helicase and RdRp domains in ORF1 and four capsid proteins in ORF2. The position of the protease domain was determined by searching for the conserved protein sequence ‘VVV QNR GSY TYH AVT FFG DCG SIL IAS NAA ITQ KIM GMH IAG ITH MNKG’ for this region [36] at residues 1395–1443 of the ORF1 protein sequence when translated from the genomic RNA. A schematic of the ALPV genome and the mapping depth is shown in Fig. 2.

### Phylogenetic and sequence comparisons between different ALPV sequences

**Fig. 3:**
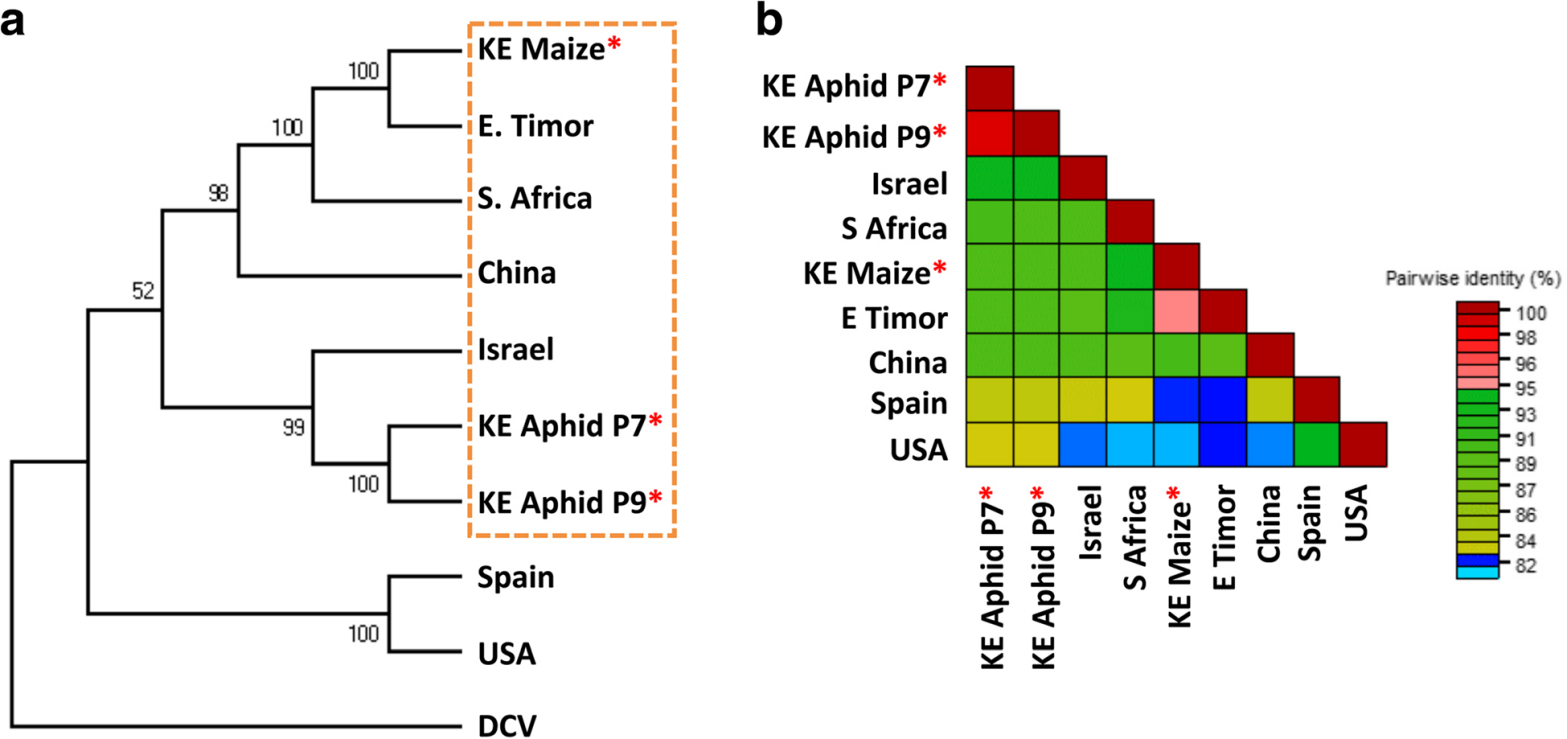
Phylogenetic analyses and pairwise sequence similarity comparisons of *Aphid lethal paralysis virus* (ALPV). **a** ALPV isolates identified from aphids in Kenya (KE) this study (KE Aphid P7 and KE Aphid P9) and an isolate from this study found in maize plants in Kenya (KE Maize) were analysed alongside six other ALPV genomic sequences available at GenBank (from China, South Africa, Israel, East Timor USA and Spain). Analysis revealed two main clades. The isolates KE Aphid P7 and KE Aphid P9 clustered in a subclade together with the ALPV isolate from Israel in the larger clade and KE Maize clustered with isolates from China, East Timor and South Africa (orange dashed box). *Drosophila C virus* (DCV) was used as the out-group. **b** The arrangement of the sequences in the pairwise comparison is based on a neighbour-joining tree from the aligned sequences. Pairwise alignments revealed similarities as low as 82% when isolates in different clades are compared. In both analyses, the sequences were aligned using MUSCLE. The phylogenetic tree was constructed using the Neighbor-Joining method with 1000 bootstraps in MEGA6. Asterisks indicate ALPV isolates identified in this study

Three ALPV genomic RNA sequences from this study and six others from GenBank were used for phylogenetic analysis (Table 2). Results revealed two main clades (Fig. 3a). The isolates from our study (KE Aphid P7, KE Aphid P9 and KE Maize) were in one of the major clades, grouping with samples from China, South Africa, and East Timor. The two isolates from the USA and Spain formed the other clade. Analyses of nucleotide sequence similarity revealed genetic variation within the subclades and between the clades detected in the phylogenetic analysis. The KE Aphid P7 and KE Aphid P9 ALPV isolates shared 98.9% nucleotide sequence identity with each other and 93% similarity to the isolate from Israel when whole genomes were compared. When compared to other isolates in the subclade (KE Maize, S. Africa, China and E. Timor), sequence similarity was about 89%. Similarity to US and Spanish isolates was 82%. The USA and Spanish isolates shared 94% nucleotide sequence similarity (Fig. 3b).

When we compared the similarity of ORF1 and ORF2 sequences from the genomic RNA of the ALPV isolates we found out that KE Aphid P7 and KE Aphid P9 shared 98.6% and 99.3% similarity in ORF1 and ORF2, respectively. When the two isolates were compared to KE Maize, the similarity was about 90.3% and 86% in ORF1 and ORF2 respectively. All three isolates from Kenya had least similarity to the isolates from Spain and the USA (approximately 83%) in both ORFs (Additional file 12: Table S5).

### Recombination analyses of ALPV ORFs

Analysis of the ORF1 and ORF2 nucleotide sequences using six different algorithms showed evidence of recombination in ALPV. ORF1 showed evidence for three recombination events (Table 3). Event 1 identified three isolates (Israel, KE Aphid P7 and KE Aphid P9) as recombinant. In this event, isolate KE Maize was identified as the most probable major parent alongside an unknown minor parent. Event 2 identified the E. Timor isolate as recombinant and KE Maize was identified as the most probable major parent alongside an unknown minor parent. Event 3 identified the isolate from China as recombinant with isolates KE Aphid P9 and E. Timor as the most probable major and minor parents respectively. ORF 2 showed one recombination event in the isolate from China isolate and KE Aphid P9 and E. Timor as the most probable major and minor antecedents, respectively.

**Table 3:** Recombination events identified by the RDP4 program

		Breakpoint position			Potential Parental sequences		Score for five detection methods in RDP4				
RNA segment	Event	Begin	End	Recombinant sequences	Major	Minor	RDP	GENECOV	MaxChi	Chimaera	SiScan
ORF 1	1	9	880	ISRAEL	KE Maize	Unknown	7.1e-38	6.4e-38	2.7e-13	3.1e-20	1.7e-28
	1	9	880	KE Aphid P7	KE Maize	Unknown	7.1e-38	6.4e-38	2.7e-13	3.1e-20	1.7e-28
	1	9	880	KE Aphid P9	KE Maize	Unknown	7.1e-38	6.4e-38	2.7e-13	3.1e-20	1.7e-28
	2	262	861	E. Timor	KE Maize	Unknown	6.8e-07	4.1e-06	1.4e-04	6.2e-05	6.1e-14
	3	879	3192	CHINA	S. Africa	Spain	6.4e-05	–	2.4e-22	1.6e-17	7.2e-68
ORF 2	1	130	645	CHINA	KE Aphid P9	E. Timor	5.9e-22	1.1e-07	6.5e-03	8.0e-12	2.1e-06

### Sequence and phylogenetic analyses reveal that BSRV-like isolates may belong to a new dicistrovirus species

From BLAST analyses, the 11 nucleotide sequences from this study had 87% similarity to isolate BSRV1 (GenBank number: JF423195.1), which is 1473 nucleotides long and encodes part of the non-structural polyprotein (ORF1) of BSRV. A BLAST analysis comparing ORF2 sequences from our BSRV-like sequences and an ORF2 sequence from GenBank, BSRV4 (GenBank number: JF423198.1) revealed 77% and 86% similarity at nucleotide and protein sequence comparisons respectively. The nucleotide similarity among the BSRV-like viruses was 98–99%.

**Fig. 4:**
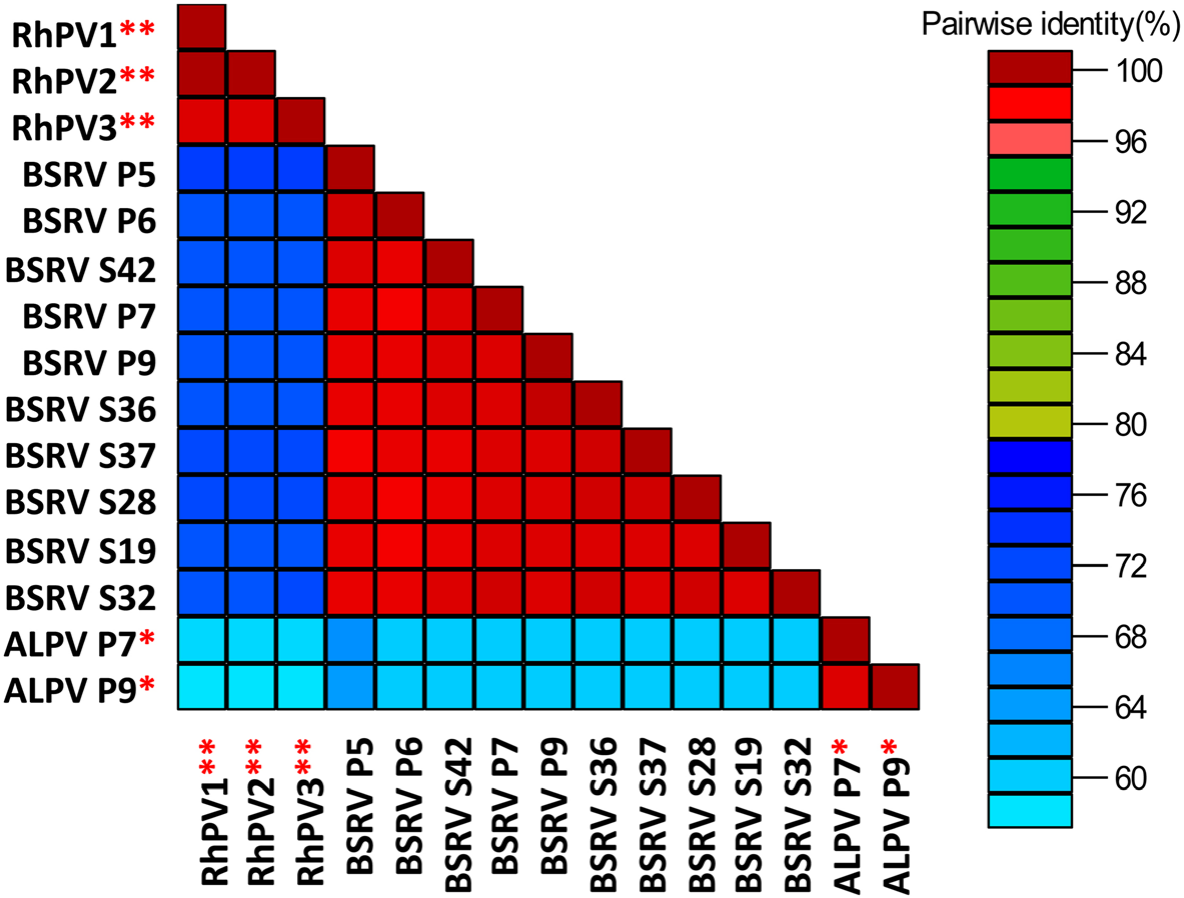
Pairwise comparison of Big Sioux River virus (BSRV)-like sequences to ALPV and RhPV sequences. The BSRV-like sequences shared up to 99% sequence similarity among each other. Similarly, the other species in the analysis showed close intra-species similarity. Interspecies comparisons revealed a clear demarcation between the different species. The sequences were aligned using MUSCLE and the arrangement of the samples in the matrix is based on phylogenetic clustering of neighbour-joining tree constructed from the multiple sequence alignment. RhPV sequences (marked with two asterisks) used in this analysis were from GenBank while the ALPV sequences used (marked with a single asterisk) were from this study

**Fig. 5:**
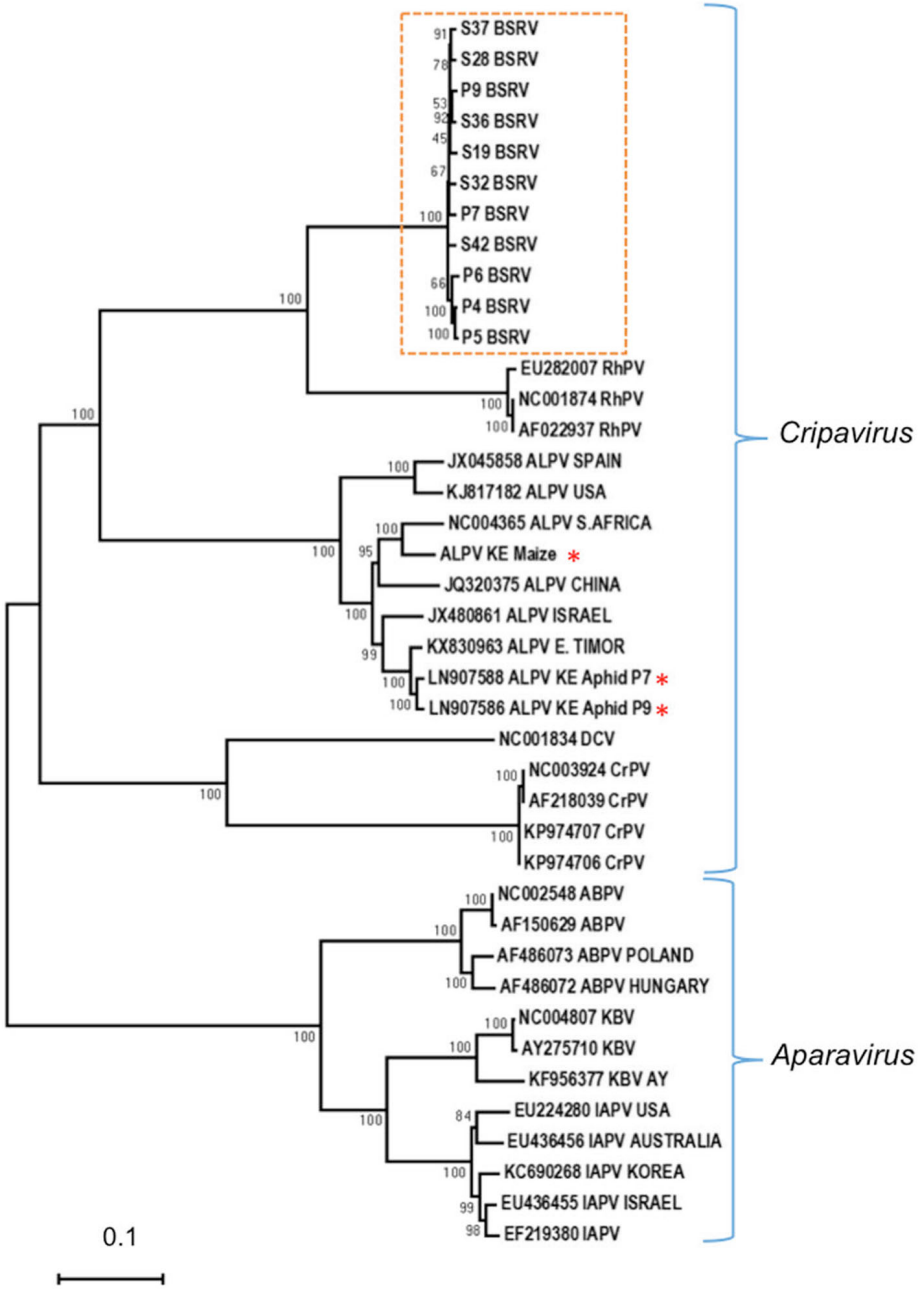
Phylogenetic analysis of Big Sioux River virus (BSRV)-like sequences identified in this study. The BSRV-like sequences from our study are (in the dashed box) clustered most closely with *Rhopalosiphum padi* virus (RhPV) in the genus *Cripavirus* [57], which include the type species *Cricket paralysis virus* (CrPV), RhPV, as well as *Aphid lethal paralysis virus* (ALPV) and *Drosophila C virus* (DCV). The second clade shown has sequences from the genus *Aparavirus* [57] including the type species *Acute bee paralysis virus* (ABPV), *Kashmir bee virus* (KBV), and *Israeli acute paralysis virus* (IAPV). The phylogenetic tree was constructed using the Neighbour-Joining method with 1000 bootstraps. ALPV isolates identified in this study are indicated with red asterisks

Comparison of the 11 BSRV-like sequences to two ALPV (KE Aphid P9, KE Aphid P7) and three RhPV sequences from GenBank revealed 57–60% similarity to ALPV and 70–72% to RhPV. Among the BSRV-like sequences the similarity was 98–99% (Fig. 4). In phylogenetic analyses, the BSRV-like sequences clustered in a clade containing members of the genus Cripavirus (Fig. 5). This demarcation confirmed that the BSRV-like isolates detected in our samples were distinct viruses unrelated to ALPV or RhPV and were likely to be a novel BSRV-like dicistrovirus.

**Fig. 6:**
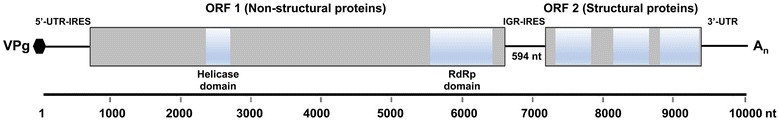
Deduced genome organization of the (BSRV)-like virus. The positive-sense single stranded RNA genome of the BSRV-like virus is approximately 10.2Kb and 3′-polyadenylated. The 5′-untranslated region (5′-UTR) and intergenic region (IGR) are 680 and 594 nucleotides (nt) long, respectively, and are internal ribosome entry sites (IRESs) for translation of open reading frames (ORFs) 1 and 2 (gray boxes). A VPg molecule is likely attached to the 5’end of the genomic RNA. The IGR-IRES for this virus is longer than that of other dicistroviruses. By comparison, RhPV is the only other dicistrovirus with a long IGR-IRES (533 nt), while the ALPV IGR-IRES is 197 nt long (see Fig. 2). ORF1 (6024 nt) is predicted to encode a non-structural protein precursor of 2008 amino acid residues. ORF2 is 2262 nucleotides long and is translated into a 754-amino acid long structural protein precursor and if consistent with other dicistroviruses, self-cleaves into mature capsid proteins. Locations of putative helicase and RdRp domains encoded by ORF1 and three putative structural proteins encoded by ORF2 (shaded blue) were obtained by searching for homology in the NCBI conserved domain database. As in Fig. 2, only those putative functional domains in the encoded proteins that can be deduced with a high degree of certainty are shown

The sequencing of BSRV-like RNA revealed two ORF. The 5′ proximal ORF 1 was predicted to encode a translation product of 2007 amino acids while ORF2 was predicted to encode a product of 753 amino acids (Fig. 6). Analysis of the genomic RNA sequence for ORF1 in the NCBI conserved protein database predicted regions with similarity to helicase and RdRp domains. The ORF2 sequence was predicted to encode a precursor for three structural proteins (Fig. 6). The length of the intergenic region of the BSRV-like viruses was 594 nucleotides long, which is longer than in any other previously reported dicistroviruses. Only protein functional domains predicted with the highest confidence are shown in the upper panel of Fig. 6.

## Discussion

In this article, we describe DNA-based species identification of winged aphids collected from farms in Kenya and the detection, using viral metagenomics, of dicistrovirus sequences in these aphids and in maize leaf samples. We found that *A. fabae* was the only aphid species present in the bean plots sampled, despite these being located on small farms that had mixed cropping systems. The lack of aphid diversity was surprising, given the diversity of plants present on the farms. For example, at the Ndeiya sampling site, beans were intercropped with maize and potato, and a wide variety of fruit trees and seasonal vegetable crops such as cabbage and amaranth were present in adjoining plots. The absence of aphids other than *A. fabae* could be due to two reasons. Firstly, the sampled plots may have had crop combinations that contained plant hosts unfavourable for other aphid species. Secondly, there may have been a seasonal effect on aphid diversity. Literature on the seasonal variation of aphids, in East Africa and Kenya specifically, is scant but past surveys conducted in Kenyan bean farms found that *A. fabae* and *A. gossypii* were the most prevalent aphids over the April to June long rainy season [37, 38] but no information was previously available for the October to November short rainy season.

Viral metagenomic analysis of aphids and maize leaf samples detected three dicistroviruses: ALPV, RhPV, and a novel BSRV-like virus. ALPV and RhPV have been detected in *R. padi*, *A. fabae* and maize in South Africa [11, 12], but not previously in East or Central Africa. The discovery of BSRV-like viruses in *A. fabae* is, to our knowledge, the first report from Africa. Phylogenetic comparison of the ALPV isolates from our study with those identified by others and deposited in GenBank revealed two main clades. These results are consistent with the analysis of Liu and colleagues [39], who also found two main clades for ALPV and suggested classification of ALPV isolates into two new species. Viral species demarcation is normally contingent on three criteria: evidence of different natural hosts; serological differences, and amino acid sequence identity between the capsid proteins being less than 90% [40]. The Spanish and US isolates that are phylogenetically in a separate clade from other ALPV isolates are the best candidates for reclassification but they fulfill only the third criterion. In our study, isolates obtained in Kenya from maize and *A. fabae* showed genetic variation, indicating that there could be more than one lineage of ALPV. However, the Kenyan isolates did not meet the divergence thresholds for reclassification as separate species.

Recombination is a powerful driver of viral speciation, especially in viruses with monopartite genomes [41, 42]. For viruses with multipartite genomes, recombination can occur more in one of the genomic RNA components though the overall rate of recombination is infrequent [43, 44]. Our sequence analyses have revealed the first evidence of recombination among for ALPV. The results point to the likelihood that the three isolates (KE Aphid P7, KE Aphid P9 and Israel) are separate strains from isolate KE Maize and this difference was caused by a recombination event.

There is currently no full genome for BSRV in GenBank and, therefore, our isolates may be BSRV or could be a BSRV-like, novel dicistrovirus species infecting *A. fabae*. The BSRV-like sequences from Kenya cluster phylogenetically with viruses of the genus *Cripavirus*. From previous reports, BSRV appears to be a multi-host pathogen; having been previously detected in honeybees, mosquitoes and soybean aphids [16–18]. All discoveries of BSRV have been through metagenomic studies and hence BSRV is yet to be formally classified by the International Committee on Taxonomy of Viruses (ICTV), which until recently has required evidence of biological properties such as pathogenicity, host range and epidemiology. However, due to the increasing rate of discovery of novel viruses by metagenomics, the ICTV is waiving these requirements in favor of phenotypes inferred from genome analysis and sequence relatedness inferred from phylogeny, homology detection and divergence metrics [45].

Dicistroviruses, such as ALPV and RhPV, utilize plants (in which they do not replicate) as reservoirs (‘vectors’) to infect new insect hosts in which these viruses can replicate [3]. There is no evidence (from experiments done using RhPV) that dicistroviruses replicate in plant tissue [10, 46]. However, experiments in barley show that RhPV travels through the plant vasculature to all parts of the plant including the roots within 7 days of inoculation [23]. On barley, *R. padi* individuals infected with RhPV have decreased fecundity and diminished behavioral responses to semiochemicals, most notably for methyl salicylate, a semiochemical that denotes host plant suitability for aphid colonization [47]. It was also noted that RhPV-infected aphids were more sensitive to the aphid alarm pheromone (*E*)-β-farnesene [47]. This semiochemical promotes dispersal, which translates to decreased aphid populations on plants. These authors also noted that aphids infected with RhPV were more frequently attacked by the predatory lady beetle *Coccinella septempunctata*, and the parasitoid wasp *Aphidius ervi* [47].

The permissiveness of plants to dicistroviruses is suggestive that plants may have evolved to enable them to benefit from the insect-lethal properties of these viruses. Aphid populations increase on common bean when the plants flower and their aggregation around flowers and developing pods causes direct feeding damage as well as virus transmission [48]. We speculate that dicistroviruses could potentially play a natural role in protecting plants through the control of aphid populations. The payback to the dicistroviruses would likely be access to new insect hosts. This conjectured mutualistic plant-virus relationship raises the possibility that plants exploit dicistroviruses as natural biopesticides.

Is there a role for beneficial insects such as pollinators in the spread of aphid-lethal dicistroviruses? ALPV has been detected in honeybees but its presence was not associated either with colony collapse disorder or any other pathology, suggesting that bees are latent hosts [16, 49]. It has been demonstrated that the honeybee-infecting dicistrovirus *Israeli acute paralysis virus* can be transmitted to non-infected individuals or colonies via virus-contaminated pollen collected during foraging on flowers [50]. Bee-infecting viruses such as *Black queen cell virus*, *Deformed wing virus* and *Chronic bee paralysis virus* have been detected in honeybee feces [51, 52]. Thus, one might speculate that pollinators may also disseminate aphid-lethal dicistroviruses.

## Conclusions

Using deep sequencing we have detected the presence of strains of three dicistroviruses, ALPV, RhPV, and a novel BSRV-like dicistrovirus, in insect (*A. fabae*) and plant (*Z. mays*) samples. This is the first report of these viruses being isolated from both organisms and the first report of these viruses in East Africa. The work indicates that for these three dicistroviruses the insect host and plant reservoir ranges as well as the extent of their geographic ranges are wider than previously suspected.

## Additional files

Additional file 1: Table S1. Metadata for aphid and maize sampling sites. (XLSX 13 kb)

Additional file 2: Text file S1. RhPV sequences from maize samples. (TXT 7kb))

Additional file 3: Table S2. List of primers used for RT-PCR and sequences generated. (XLSX 18 kb)

Additional file 4: Text file S2. *CO1* sequences from Sanger sequencing. (TXT 24 kb)

Additional file 5: Text file S3. *CO1* sequences from Virfind. (TXT 43 kb)

Additional file 6: Table S3. BLAST results for aphid identification by *CO1* gene from Sanger sequencing and Virfind. (XLSX 12 kb)

Additional file 7: Text file S4. ALPV sequences from maize samples. (TXT 23 kb)

Additional file 8: Text file S5. RhPV sequences from aphid samples. (TXT 4 kb)

Additional file 9: Table S4. Virus-like sequences from BLASTx. (XLSX 13 kb)

Additional file 10: Fig.S1. Confirmation of the presence of *Aphid lethal paralysis virus* (ALPV) and a Big Sioux River-like virus. (PDF 1.5 MB)

Additional file 11: Fig. S2. RT-PCR confirmation of *Rhopalosiphum padi* virus in maize samples collected from Kitui County. (PDF 84 kb)

Additional file 12: Table S5. ALPV and BSRV-like virus Pairwise comparisons. (XLSX 12 kb)
