# Supplementary material for: Viral metagenomics of aphids present in bean and maize plots on mixed-use farms in Kenya reveals the presence of three dicistroviruses including a novel Big Sioux River virus-like dicistrovirus
Source: Virol J. 2017 Oct 2;14:188. doi: 10.1186/s12985-017-0854-x (PMC5625602; doi:10.1186/s12985-017-0854-x)
Supplement: Supplementary file 10 — Confirmation of the presence of Aphid lethal paralysis virus (ALPV) and a Big Sioux River-like virus. (PDF 1.5 MB) [file 12985_2017_854_MOESM10_ESM.pdf]

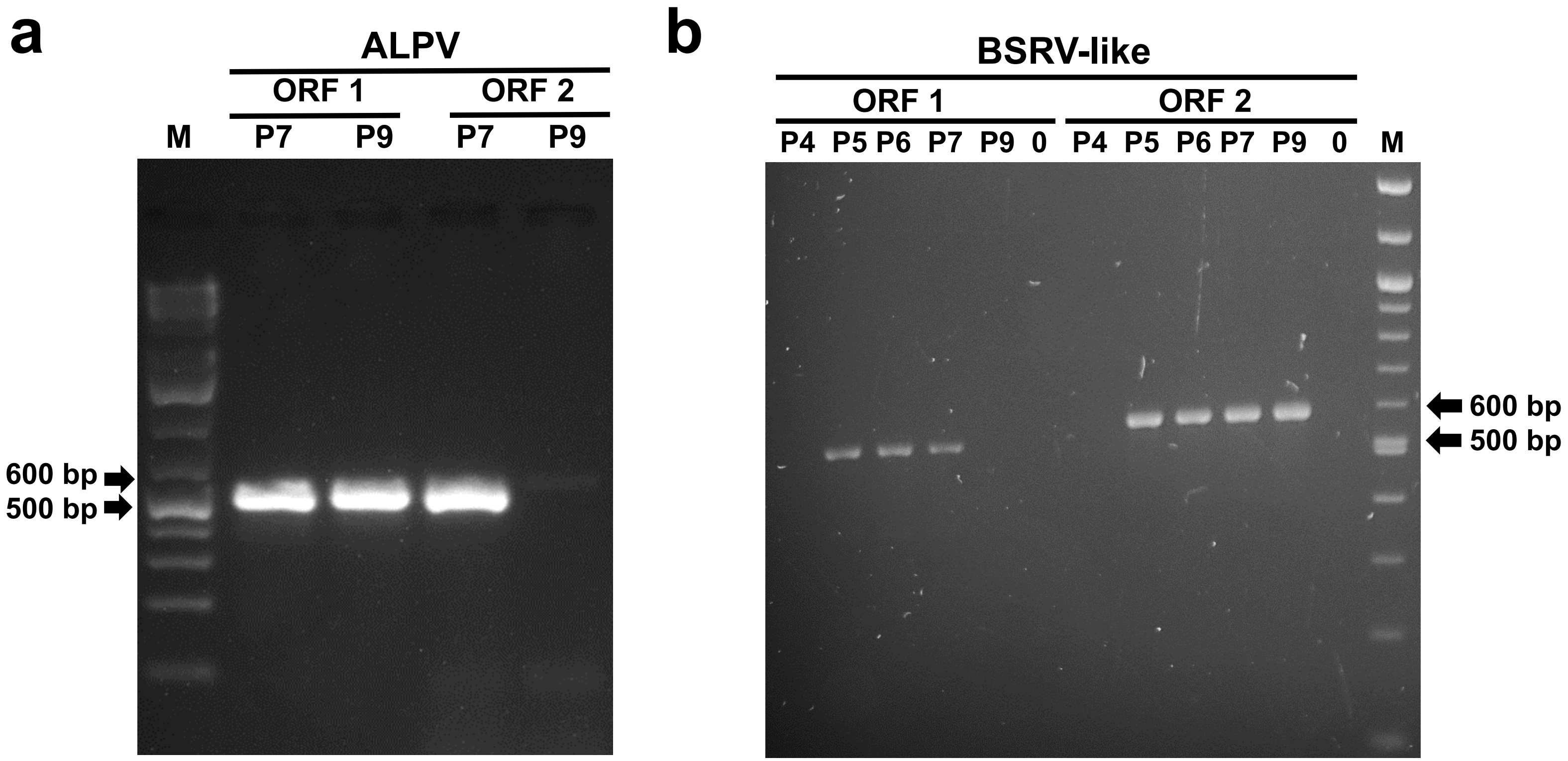

Additional File 10: Fig. S1 Confirmation of the presence of *Aphid lethal paralysis virus* (ALPV) and a Big Sioux River-like virus. RT-PCR using primers specific for open reading frames (ORFs) 1 and 2 of ALPV (**a**) and the BSRV-like virus (**b**) was carried out on a selection of aphid RNA samples (see main text for descriptions of samples P4 through P9) to confirm viral metagenomic analyses. PCR amplification products were analyzed by electrophoresis on a 2% agarose gel and visualized by ethidium bromide staining and UV illumination. In all cases, 550 base-pair (bp) was the size expected for the amplified DNA products. Lanes marked M were loaded with DNA size markers and positions of the 500 and 600 bp markers are indicated. The lane marked ‘0’ was loaded with a control PCR in which water was substituted for cDNA template.
