# Supplementary material for: Viral metagenomics of aphids present in bean and maize plots on mixed-use farms in Kenya reveals the presence of three dicistroviruses including a novel Big Sioux River virus-like dicistrovirus
Source: Virol J. 2017 Oct 2;14:188. doi: 10.1186/s12985-017-0854-x (PMC5625602; doi:10.1186/s12985-017-0854-x)
Supplement: Supplementary file 11 — RT-PCR confirmation of Rhopalosiphum padi virus in maize samples collected from Kitui County. (PDF 84 kb) [file 12985_2017_854_MOESM11_ESM.pdf]

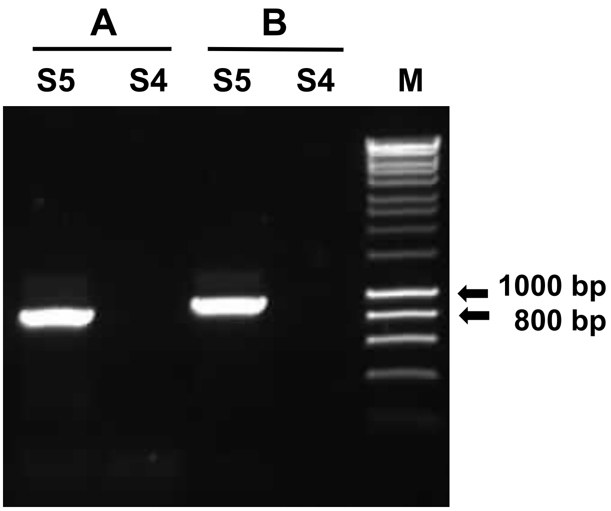

Additional File 11 Fig. S2 RT-PCR confirmation of *Rhopalosiphum padi* virus in maize samples collected from Kitui County. RT-PCR, using two independent primer sets specific for *Rhopalosiphum padi* virus (RhPV), was used to confirm the presence of RhPV in two samples (T2F3S4 (abbreviated as S4) and T2F2S5 (abbreviated as S5) where RhPV had been detected following *de novo* sequence assembly in Trinity. PCR amplification products were analyzed by electrophoresis on a 2% agarose gel and visualized by ethidium bromide staining and UV illumination. The expected product size for amplified DNA products using primer pair RPV_200_F1 / RPV_1100_R1 (A) was 789 base-pairs (bp) while that of RPV_1350_F1/ RPV_ 2300_R1 (B) was 836 bp. Lane M was loaded with DNA size markers and positions of the 800 and 1000 bp markers are indicated.
